# Reductions in serum IGF-1 during aging impair health span

**DOI:** 10.1111/acel.12188

**Published:** 2013-12-30

**Authors:** Zhenwei Gong, Oran Kennedy, Hui Sun, YingJie Wu, Garry A Williams, Laura Klein, Luis Cardoso, Ronald W Matheny, Gene B Hubbard, Yuji Ikeno, Roger P Farrar, Mitchell B Schaffler, Martin L Adamo, Radhika H Muzumdar, Shoshana Yakar

**Affiliations:** 1 Department of Pediatrics, Albert Einstein College of MedicineBronx, New York, 10461, USA; 2 Department of Orthopaedic Surgery, New York University, Hospital for Joint DiseasesNY, NY, 10003, USA; 3 David B. Kriser Dental Center, Department of Basic Science and Craniofacial Biology, New York University College of DentistryNew York, NY, 10010, USA; 4 Department of Biomedical Engineering, The City College of New YorkNew York, NY, 10031, USA; 5 Department of Biochemistry, University of Texas Health Science CenterSan Antonio, TX, 782297, USA; 6 Sam and Ann Barshop Institute for Longevity and Aging Studies, University of Texas Health Science CenterSan Antonio, TX, 78229, USA; 7 Department of Pathology, University of Texas Health Science CenterSan Antonio, TX, 78229, USA; 8 Department of Kinesiology and Health Education, University of Texas at AustinAustin, TX, 78712, USA; 9 College of Integrative Medicine and Institute of Integrative Medicine, Dalian Medical UniversityDalian, China; 10 US Army Research Institute of Environmental Medicine, Military Performance DivisionNatick, MA 01760, USA

**Keywords:** aging, bone, growth hormone, IGF, insulin-sensitivity, LID, lifespan, liver, tumor

## Abstract

In lower or simple species, such as worms and flies, disruption of the insulin-like growth factor (IGF)-1 and the insulin signaling pathways has been shown to increase lifespan. In rodents, however, growth hormone (GH) regulates IGF-1 levels in serum and tissues and can modulate lifespan via/or independent of IGF-1. Rodent models, where the GH/IGF-1 axis was ablated congenitally, show increased lifespan. However, in contrast to rodents where serum IGF-1 levels are high throughout life, in humans, serum IGF-1 peaks during puberty and declines thereafter during aging. Thus, animal models with congenital disruption of the GH/IGF-1 axis are unable to clearly distinguish between developmental and age-related effects of GH/IGF-1 on health. To overcome this caveat, we developed an inducible liver IGF-1-deficient (iLID) mouse that allows temporal control of serum IGF-1. Deletion of liver *Igf* -*1* gene at one year of age reduced serum IGF-1 by 70% and dramatically impaired health span of the iLID mice. Reductions in serum IGF-1 were coupled with increased GH levels and increased basal STAT5B phosphorylation in livers of iLID mice. These changes were associated with increased liver weight, increased liver inflammation, increased oxidative stress in liver and muscle, and increased incidence of hepatic tumors. Lastly, despite elevations in serum GH, low levels of serum IGF-1 from 1 year of age compromised skeletal integrity and accelerated bone loss. We conclude that an intact GH/IGF-1 axis is essential to maintain health span and that elevated GH, even late in life, associates with increased pathology.

## Introduction

The role of growth hormone (GH)/insulin-like growth factor-1 (IGF-1) axis in lifespan has been studied in detail along the evolutionary lineage. Disruption of insulin/IGF-like signaling in yeast, worms, and flies has been conclusively shown to increase lifespan. In these lower order species, insulin and IGF-1 axis exists as a single axis. However, in rodents and other mammalian species, the insulin and IGF-1 axes are complex and distinct even though they share homology. In mammals, GH regulates serum IGF-1 levels, and while the insulin axis predominantly influences metabolism, the GH/IGF-1 axis is concerned with cell survival and growth.

The evidence for the role of IGF-1 in health/lifespan in primates is even less conclusive (Colman *et al*., 2009; Mattison *et al*., 2012; Stein *et al*., 2012). In humans, serum GH concentrations and GH secretion rates fall in adulthood, accompanied by a gradual fall in plasma IGF-1 concentrations, a state often termed the somatopause. In humans, reductions in IGF-1 also associate with higher incidence of diabetes (Barzilai *et al*., 2012), osteoporosis (Lombardi *et al*., 2005), dementia, and Alzheimer disease (O’Neill *et al*., 2012). Decline in GH/IGF-1 axis has been also shown to be associated with decreased incidence of tumors (Bartke *et al*., 2013). Indeed, human subjects with mutations in the GHR (Laron dwarfs) who show congenital IGF-1 deficiency do not exhibit increases in lifespan, but show protection from development of malignancies and diabetes when compared to their healthy siblings (Laron, 2005; Guevara-Aguirre *et al*., 2011).

Rodent models where global IGF-1 is decreased, such as caloric restriction (Wolf *et al*., 2000; Ward *et al*., 2005), and GH receptor deletion in mice (Hauck & Bartke, 2000; Westbrook *et al*., 2009) show increased lifespan. On the other hand, mice overexpressing GH throughout their lifespan live shorter (Bartke *et al*., 2002; Sonntag *et al*., 2012). Although the aforementioned studies point to a simple cause and effect relationship between the GH/IGF-1 axis and lifespan, experiments in many rodent models point to more complex interactions. The latter may depend on species, genetic background, gender, or even husbandry conditions. For example; female mice with *igf-1 receptor* (*igfr*) haploinsufficiency lived longer than control females in one study (Holzenberger *et al*., 2003), but when repeated, the effect was smaller (Bokov *et al*., 2011). In addition to these studies, a brain-specific knockout of the *Igf-1r* was associated with increased median lifespan. However, these animals also show reduced GH levels, and thus, the effects of the *Igf-1r* deficiency cannot be distinguished from those of reduced GH levels (Kappeler *et al*., 2008). Furthermore, animal models in which components of the IGF-1 receptor signaling pathway were ablated, exhibit no change, or even reduced lifespan (Taguchi *et al*., 2007; Selman *et al*., 2008) as compared to controls.

While studies in rodents provide significant insights into the GH/IGF system and its role in aging, interpretations of the observations are complicated due to (i) developmental effects secondary to loss of the GH/IGF axis during crucial phases of growth and development and (ii) difficulties in distinguishing effects of serum IGF-1 from tissue IGF-1, and those arising from secondary increases in GH.

To better address how decline in serum IGF-1 affects aging, we used two mouse models: (i) congenital liver IGF-1-deficient (LID) mice, where serum IGF-1 is low throughout lifespan, and (ii) an inducible liver *Igf1* gene-deleted (iLID) mouse model (Wu *et al*., 2010; Courtland *et al*., 2011), which allows temporal control of IGF-1. The iLID mice express the Cre recombinase under the liver-specific ET (tamoxifen-inducible)-α1-antitrypsin promoter, and induction of the Cre recombinase was achieved by a single intraperitoneal injection of tamoxifen.

Using the congenital LID cohort, we performed survival studies and end-of-life pathology. Using the iLID model, we depleted serum IGF-1 levels when mice reached 1 year of age and followed them up to 2 years of age. Unfortunately, we did not perform survival studies in the iLID cohort, but at 2 years, we studied inflammation, oxidative stress resistance, tumor incidence, and bone loss.

## Results

### Congenital decreases in serum IGF-1 impair lifespan in male mice

**Figure 1:**
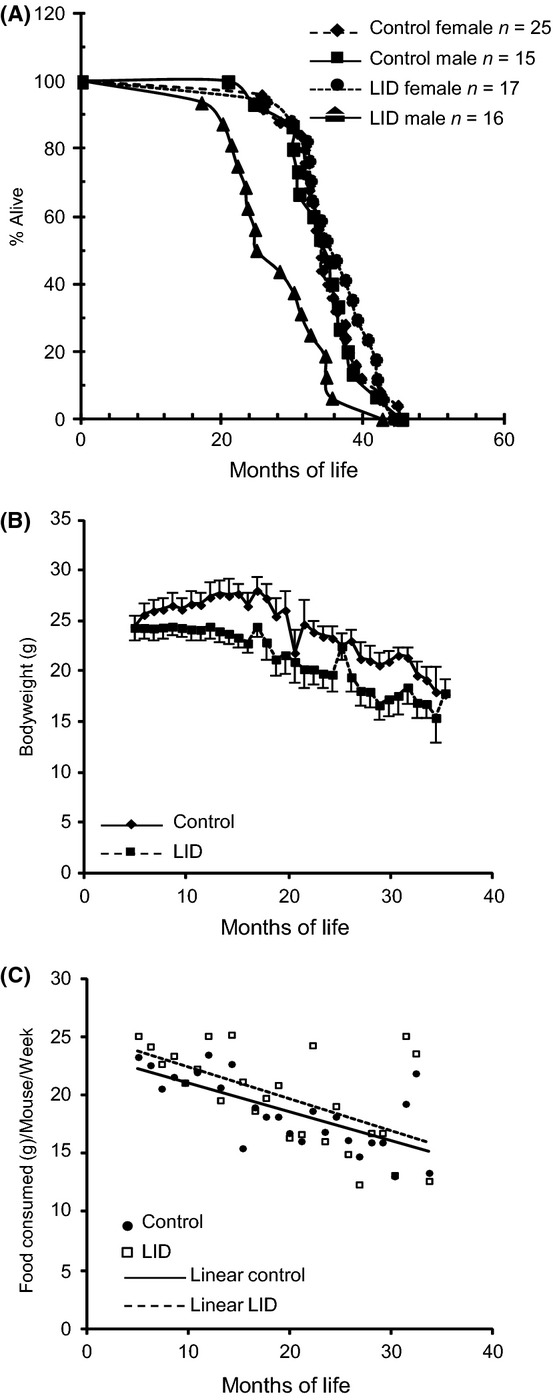
Kaplan–Meier product limit estimates of the survival curves of male and female control and LID mice. (A) Female LID mice (n = 17) show no significant difference in the survival from female controls (n = 25). However, LID males (n = 16) live shorter than male controls (n = 15), *P* = 0.006; quantile regression indicates that most of the effect occurs in first half of lifespan; means are significantly different. (B) Body weight and (C) food intake in male mice determined at 2 weeks intervals.

To understand how reductions in serum IGF-1 levels affect overall lifespan, we used the LID mouse model, where the Cre recombinase was expressed under the albumin promoter, specifically in the liver (Yakar *et al*., 1999). LID mice show early postnatal gene recombination (1 week old) and exhibit reductions in serum IGF-1 levels throughout life with concomitant increases in serum GH levels. Male and female (control and LID) mice were aged in the Nathan Shock Center at Texas Health Science Center, San Antonio (UTHSCSA). We observed robust longevity in both control male (median lifespan of 34.7 ± 1.3 months) and control female (median lifespan of 34.8 ± 1.0 months) mice. The overall survival, as analyzed by the log-rank test, was similar in female LID (with median lifespan of 36.2 ± 1.2 months). In contrast, overall survival of male LID mice was 27.9 ± 1.7 months, which was statistically significantly lower (*P* = 0.006) than the other groups (Fig. 1A, Table S1). Male LID mice showed decreased body weight throughout life (Fig. 1B) with similar food intake (Fig. 1C) when compared to controls.

Tissues dissected from a subcohort of male LID and control mice at 19-20 months of age (Table S2) revealed significant increases in absolute and relative liver weight in LID mice. Additionally, we found that absolute and relative kidney weight and quadriceps weight were significantly reduced in LID mice when compared to controls.

The probable causes of death for the LID and control groups are shown in Table 1. Approximately 22% of male control and 55% of female control mice died from neoplastic diseases. The major fatal neoplastic diseases observed in these control mice were hepatocellular carcinoma (HCC), pulmonary adenocarcinoma, and pituitary adenoma (female mice only). In addition, the neoplastic diseases were usually associated with metastasis to the other organs or other pathological lesions, for example pleural effusion, ascites, hemorrhage in the pleural and/or abdominal cavities, and severe congestion and edema in the lung. Male LID mice showed a higher incidence of fatal neoplasms (approximately 69%) than their control mice, although the changes in incidence were not statistically significant. The incidence of fatal HCC was significantly higher in male LID mice (approximately 69%) than the control group (5.6%; *P* < 0.05). The female LID mice showed a similar incidence of fatal neoplasms compared with their control mice. Although the incidence of fatal HCC was slightly higher in female LID mice (approximately 24%) than in the control group (4.5%), the difference was not statistically significant. The major fatal non-neoplastic diseases observed in these mice were glomerulonephritis, thrombus in heart, hydronephrosis, and acidophilic macrophage pneumonia. These lesions were usually associated with other pathological lesions, for example pleural effusion, ascites, and/or severe congestion and edema in the lung. The incidence of fatal glomerulonephritis was slightly lower in both male and female LID mice than in the control group; however, the difference was not statistically significant.

**Table 1:** End-of-life pathology of control and LID mice aged in the Nathan Shock Center at Texas Health Center, San Antonio (UTHSCSA)

	Male		Female	
	Control (n = 18)	LID (n = 16)	Control (n = 22)	LID (n = 17)
Neoplasm	4 (22.2%)	11 (68.8%)	12 (54.5%)	10 (58.8%)
Hepatocellular carcinoma	1 (5.6%)	11 (68.8%)*	1 (4.5%)	4 (23.5)
Adenocarcinoma	3	0	1	3
Pituitary adenoma	0	0	2	1
Others	0	0	8	2
Non-neoplasm	14	5	10	7
Glomerulonephritis	6	1	8	2
Acidophilic mac.pn.	1	3	0	0
Others	3	0	1	2
Undetermined	4	1	1	3

* *P* < 0.05.

The described studies with the LID model indicated that lifelong reductions in serum IGF-1 levels impaired health span mainly in male mice and coincided with hepatomegaly, decreased muscle and kidney weight, and increased HCC. However, one limitation of the LID mouse model is lifelong reductions in serum IGF-1; thus, the true impact of serum IGF-1 reductions after sexual maturation, during adulthood, remained unclear. To understand how reductions in serum IGF-1 during adulthood affect aging, we developed an inducible LID (iLID) model.

### Generation of a mouse model with age-dependent reductions in serum IGF-1 levels

**Figure 2:**
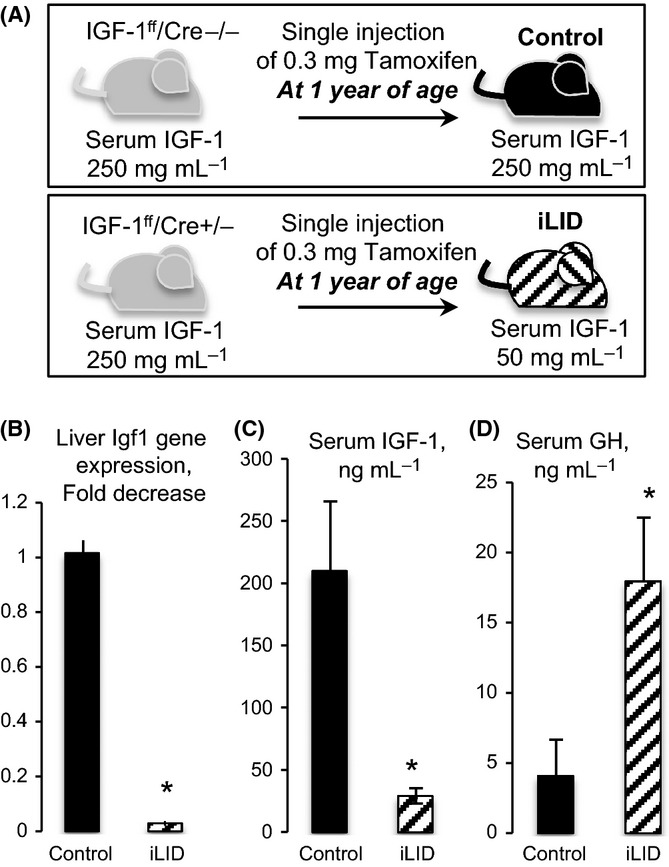
Validation of the iLID mouse model. (A) Schematic presentation of the iLID mouse model. (B) Hepatic *Igf-1* gene expression at 2 years of age in iLID (n = 5) and control (n = 5) mice following a single intraperitoneal injection of 0.3 mg of tamoxifen at one year of age. (C) Serum IGF-1 levels at 2 years of age in iLID (n = 30) and control (n = 20) mice. (D) Serum GH levels at 2 years of age in iLID (n = 10) and control (n = 9) mice. Significance (*) was considered at *P* < 0.05.

Generation of the iLID mice, described earlier (Wu *et al*., 2010; Courtland *et al*., 2011), was achieved by crossing mice homozygous for the floxed Igf-1 allele with mice expressing the Cre recombinase under the tamoxifen-inducible antitrypsin 1-α promoter specifically in the liver. Control mice were homozygous for the floxed Igf-1 allele but did not carry the Cre transgene. Ablation of hepatic Igf-1 gene was induced in iLID mice by a single intraperitoneal injection of 0.3 mg of tamoxifen at one year of age (Fig. 2A). Control mice were also injected with 0.3 mg of tamoxifen at one year of age. Mice were followed to two years of age, at which time they were sacrificed and organs dissected for further analyses and pathology.

To validate that *Igf-1* gene recombination occurred in livers of iLID mice, we assessed *Igf-1* gene expression in livers of control and iLID mice at sacrifice. Indeed, liver *Igf-1* gene expression was significantly reduced in iLID mice (Fig. 2B), leading to marked reductions in serum IGF-1 levels (Fig. 2C) and a concomitant increase in serum GH levels (Fig. 2D).

### Age-dependent reductions in serum IGF-1 are associated with increased liver weight, inflammation, and tumors

Depletion of liver *Igf-1* at ‘middle age’ (~1 year old in a mouse lifespan) in male iLID mice resulted in minor, but significant reductions in body weight and gonadal fat pad weights at two years of age (Table S3). Although a direct comparison between the LID cohort and the iLID cohort cannot be made, we found that similar to the congenital LID mice, reductions in serum IGF-1 in the iLID mice (in mid-life) associated with significant reductions in kidney weight (Tables S2, S3). Additionally, we found that relative heart weight increased in iLID mice as compared to controls (while this was not significant in the LID cohort).

**Figure 3:**
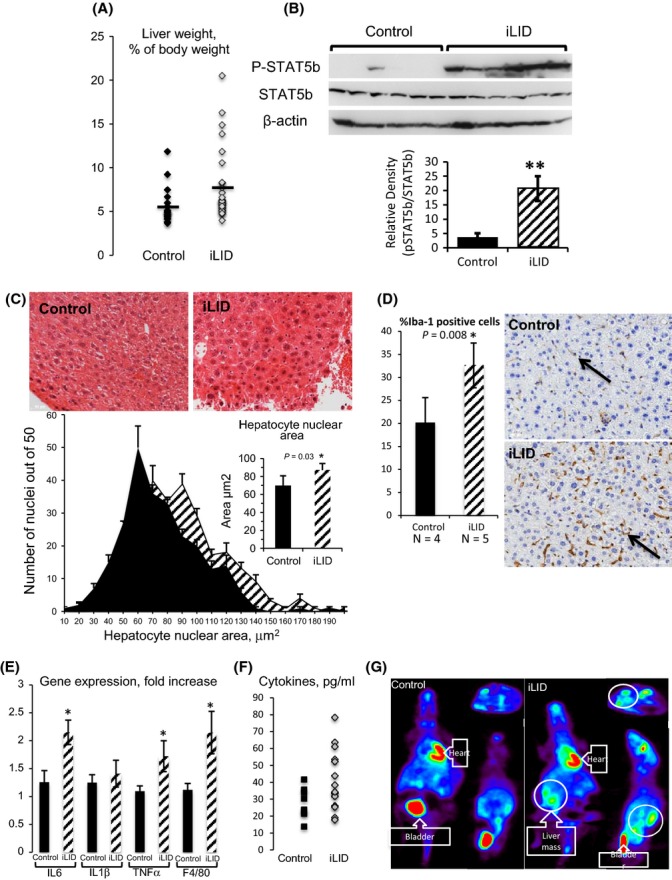
Inducible liver IGF-1-deficient mice show increased hepatic size, inflammation, and tumors. (A) Relative liver weight increased significantly in iLID mice (n = 36) as compared to controls (n = 27). (B) iLID mice (n = 10) show increased basal STAT5b-phosphorylation (pSTAT5b) as compared to controls (n = 10, *P* = 0.0015). Phosphorylated STAT5b levels were corrected to total STAT5b protein levels detected using the same membrane. (C) Nuclear size was evaluated in H&E-stained liver sections. Fifty nuclei per section were measured and calibrated to an image scale bar using Image J software. Distribution histogram shows nuclear size in control and iLID mice, and insert bar graph shows mean nuclear size (N = 5 mice per group). (D) The fraction (%) of *Iba1*-positive Kuppfer cells counted in three sections per mouse from control (n = 4) and iLID livers (n = 5 per group). iLID mice showed significant (*P* = 0.008) increase in Iba-1-positive cells in their livers. (E) Gene expression of markers of inflammation in iLID and control livers (n > 7). (F) Serum cytokine levels in control and iLID mice (graph represents overall cytokines measured in all mice, see also Table S4). (G) A representative PET scan image of iLID and control mice demonstrating increased uptake at the site of liver tumor.

Interestingly, as seen in the LID cohort, we found that iLID mice had significantly increased relative liver weight (Fig. 3A). This was associated with enhanced basal STAT5b phosphorylation (pSTAT5b) in livers of iLID mice (*P* = 0.0015), possibly due to increased serum GH levels (Fig. 3B). Furthermore, in accordance with previous publications using GH transgenic mice (Miquet *et al*., 2013), we found that aged iLID hepatocytes manifested a higher mean nuclear area when compared to controls (Fig. 3C) with a shift to larger nuclear area in the nuclear size distribution histogram.

Histological examination of liver sections revealed no increase in hepatic lipid accumulation (data not shown). However, there was an increased lymphoplasmacytic infiltrate in the portal regions in iLID mice, while no inflammatory infiltrates were found in control mice at 2 years of age (Fig. 3D). Additionally, the number of Iba1 (a marker of macrophages) positive Kupffer cells significantly increased in iLID livers (Fig. 3D). Likewise, F4/80 gene expression, a marker for macrophages, was significantly higher in iLID livers (Fig. 3E). Consistent with an increase in inflammatory infiltrate, gene expression of the cytokines IL-6 and TNFα was significantly increased in livers of iLID mice (Fig. 3E). Moreover, the profile of cytokines in serum in mice showed marginally higher levels in the iLIDs compared with controls, although no significant differences were found (Fig. 3F, Table S4).

Macroscopic (PET scan) and histological examinations indicated an increase in the incidence of hepatic tumors in the iLID mice at two years of age (Fig. 3G). The incidence of hepatic tumors was 26% in control and 42% in iLID mice, but did not reach significance. Extrahepatic tumor incidence was 11% in control and 3% in iLID mice (Table S5). In a subset of control and iLID mice, PET scans were taken to localize tumors. Figure 3G is a representative PET scan image of iLID and control mice demonstrating increased FDG uptake at the site of the tumor. The location of tumors as noted on PET scans was confirmed at the time of sacrifice.

### Age-dependent reductions in serum IGF-1 is associated with increased oxidative stress in muscle and liver

**Figure 4:**
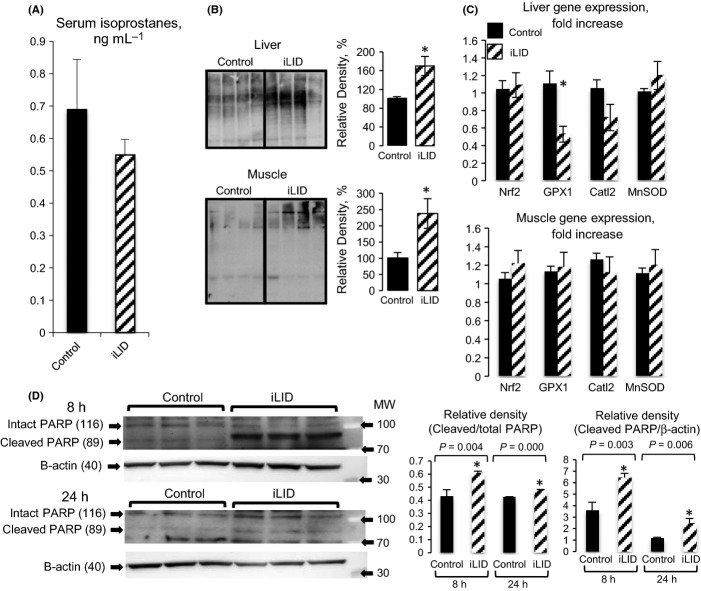
Inducible liver IGF-1-deficient mice show increased markers of oxidative stress. (A) Serum isoprostanes in control (n = 4) and iLID (n = 4) mice. (B) Carbonyl group-contained proteins, detected by OxyBlot™ Protein Oxidation Detection Kit, increased in both livers and muscles of iLID mice compared with controls (n > 4 per group). Relative density corresponds to whole lane. (C) Gene expression of markers of antioxidant defense enzymes in the livers and muscles of iLID (n = 5) and control (n = 5) mice. (D) Peroxide-induced PARP cleavage in primary skin fibroblasts isolated from 2-year-old control (n = 4) and iLID mice (n = 5). Cultures were grown to 80% confluence and treated with 400 µm peroxide for 8 and 24 h. PARP cleavage was detected by conventional Western immunoblotting. The levels of cleaved PARP were corrected to both total PARP and β-actin, detected on the same blot.

We examined how reductions in serum IGF-1 levels during aging affect reactive oxygen species (ROS) generation by assaying lipid peroxidation (8-iso-prostaglandin F2-alpha (8-isoprostane)) in serum. This assay requires large amounts of sera, and therefore, we had to pool samples. Surprisingly, we found that iLID and control mice had comparable levels of serum isoprostanes (Fig. 4A).

To better understand how hepatic Igf-1 gene deletion affected oxidative stress resistance in liver and muscle, we used the OxyBlot™ (OxyBlot Protein Oxidation Detection Kit S7150; Millipore, Billerica, MA, USA) Protein Oxidation Detection method. This assay detects carbonyl group-containing proteins that result from oxidative reactions with ozone, oxides of nitrogen, or by metal-catalyzed oxidation. We found that livers of iLID mice showed a 60% increase in oxidized proteins as compared to controls, whereas muscles of iLID mice had a twofold increase in oxidized proteins compared with controls (Fig. 4B).

Antioxidant defense includes induction of enzymatic machinery that converts superoxide to hydrogen peroxide and molecular oxygen. The Nrf2 transcription factor is a master regulator of genes that are involved in detoxification of harmful compounds such as glutathione peroxidase (Gpx1) and catalase 2 enzymes. We therefore examined the expression levels of Nrf2, GPX1, and catalase in liver and muscle of iLID and control mice at 2 years of age. We found that there were no differences in Nrf2 gene expression (assessed by 2 sets of primers) in liver and muscle from iLID mice as compared to controls (Fig. 4C). However, Gpx1 gene expression significantly decreased (60% reduction) in the livers (but not in muscle) of iLID mice, while no changes were detected in the expression levels of catalase and superoxide dismutase in the livers (Fig. 4C) or muscle between the groups.

Lastly, we assessed oxidative stress resistance of dermal fibroblasts isolated at time of dissection from 2-year-old mice. Primary fibroblast cultures were grown to 80% confluence and treated with peroxide (400uM) for 8 or 24 h. As seen in Fig. 4D, peroxide induced significantly more PARP cleavage in fibroblasts isolated from aged iLID mice as compared to controls. This was evident when the intensity of the cleaved PARP band was corrected to intact PARP or to the β-actin band intensities. All together, these data indicate that iLID fibroblasts were more sensitive to the cytotoxic effects of peroxide.

### Reductions in serum IGF-1 during aging compromise skeletal integrity

**Figure 5:**
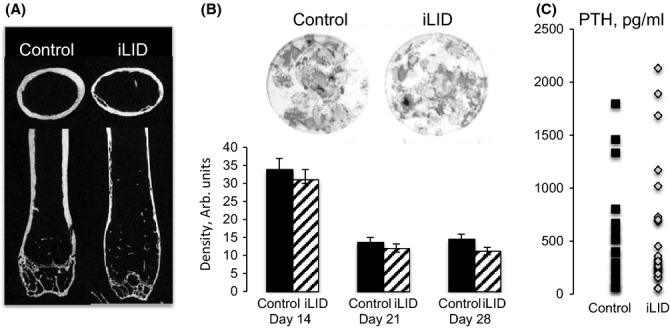
Inducible liver IGF-1-deficient mice exhibit impaired skeletal integrity during aging without changes in bone marrow osteoprogenitor number or serum PTH levels. (A) A representative image of bones from control and iLID mice injected with tamoxifen (0.3 mg) at 1 year of age and dissected at 2 years. (B) Primary osteoblast-like cultures of bone marrow mesenchymal stem cells dissected at 2 years of age. Cultures maintained in differentiation media for 14, 21 and 28 days and the density of alkaline phosphatase-positive colonies was calculated, (control n = 5, iLID n = 7). (C) Serum PTH levels in control (n = 18) and iLID (n = 26) mice at 2 years of age.

Femurs dissected from 2-year-old control and iLID mice were analyzed by micro-CT (Table 2, Fig. 5A). We found that reductions in serum IGF-1 levels in iLID mice at one year resulted in significant loss of cortical bone at two years. This was evident by reduced total cross-sectional area (Tt.Ar), cortical bone area (Ct.Ar), cortical bone thickness (Ct.Th), marrow area (Ma.Ar), and relative cortical bone area (RCA; Ct.Ar/Tt.Ar). There were minor differences in trabecular bone traits. Specifically, iLID mice showed reduced trabecular number when compared to controls and an increase in trabecular thickness. However, overall bone volume per total volume (%BV/TV) and bone mineral density (BMD) at the distal femur were comparable between the groups (Table 2). Mechanical properties of bones, dissected at 2 years, followed the morphological properties obtained by micro-CT. Four-point bending testing showed that iLID aged bones were mechanically inferior when compared to controls (Table 2). As such, max load, stiffness, yield to fracture, and postyield displacement were all significantly lower in iLID bones.

**Table 2:** Bone morphology and mechanical properties of femurs from control and iLID mice. Femurs dissected from 2-year-old mice were subjected first to micro-CT and consequently 4-point bending tests and ICP

	Control (n = 14)	iLID (n = 12)	*P*
Body weight, g	35.95 ± 4.0	30.22 ± 5.0	0.002
Cortical bone (femoral mid-diaphysis)			
Total cross-sectional area (Tt.Ar), mm^2^	2.38 ± 0.31	1.94 ± 0.24	0.000
Cortical bone area (Ct.Ar), mm^2^	0.84 ± 0.07	0.60 ± 0.08	0.000
Cortical bone thickness (Ct.Th), mm	0.14 ± 0.01	0.10 ± 0.01	0.000
Marrow area (Ma.Ar), mm^2^	1.53 ± 0.26	1.34 ± 0.17	0.044
Relative cortical area	0.35 ± 0.03	0.30 ± 0.01	0.000
Polar moment of inertia (J0), mm^4^	0.55 ± 0.11	0.35 ± 0.07	0.000
Tissue Mineral Density, g/cc	1.46 ± 0.07	1.47 ± 0.05	0.696
Trabecular bone (distal femur)			
Bone volume/Total volume (BV/TV),%	3.62 ± 2.48	2.27 ± 1.49	0.137
Trabecular thickness (Tb.Th), mm	0.04 ± 0.00	0.05 ± 0.00	0.005
Trabecular number (Tb.N), 1/mm	0.83 ± 0.51	0.43 ± 0.26	0.036
Trabecular spacing (Tb.Sp), mm	0.37 ± 0.12	0.32 ± 0.09	0.354
Bone mineral density, g/cc	0.14 ± 0.04	0.11 ± 0.02	0.158
Mechanical properties (4-point bending assay)			
Max load, N	26.67 ± 4.10	13.27 ± 4.49	0.000
Stiffness, N/mm	115.87 ± 30.47	81.19 ± 37.23	0.017
Work to fracture, Nmm	5.83 ± 3.16	1.92 ± 0.78	0.003
Postyield displacement, mm	0.256 ± 0.125	0.100 ± 0.036	0.002
Bone mineral compositions (ICP)			
Calcium (Ca^2+^), ppm	37.25 ± 0.96	37.72 ± 1.00	0.250
Phosphate (Pi), ppm	17.75 ± 0.44	18.17 ± 0.50	0.035
Pi/Ca^2+^ ratio	0.47 ± 0.00	0.48 ± 0.00	0.005
Ca^2+^/Pi ratio	2.09 ± 0.01	2.07 ± 0.00	0.005

Bone mineral composition was measured by inductively coupled plasma (ICP) assay (Table 2) in whole femurs dissected from 2-year-old mice**.** We found that iLID mice had significantly lower ratio of Ca/Pi when compared to controls, which was not reflected in tissue mineral density (TMD) of cortical bone or in trabecular BMD.

To understand whether reductions in serum IGF-1 affected the number of osteoprogenitors in the bone marrow, we established osteoblast-like primary cultures. The number of alkaline positive colonies after 14, 21, and 28 days in cultures was similar between controls and iLID mice (Fig. 5B). Osteoclast primary cultures did not differ between the groups (data not shown).

Serum osteocalcin, a marker of bone formation, did not differ between control (29.8 ± 6.9 ng mL^−1^) and iLID mice (28.1 ± 11.5 ng mL^−1^) at two years of age. Another important hormone, which regulates bone remodeling during aging is parathyroid hormone (PTH). Elevations in PTH often associate with reduced bone mass. We found that control and iLID mice show similar levels of PTH in serum (Fig. 5C).

## Discussion

In this study, we have aged two animal cohorts of congenital liver IGF-1 deficiency (LID) and an inducible LID (iLID) and studied the role of the GH/IGF-1 axis during aging. Although a direct comparison between the 2 cohorts cannot be made, we have revealed that reductions in serum IGF-1 levels in both LID and iLID mice with concomitant increases in GH associated with hepatomegaly, decreased kidney size and increased incidence of hepatic tumors. In both models, we show that in face of low levels of serum IGF-1, high levels of GH (even during aging only) associate with increased pathology.

Survival studies with the LID mouse model have revealed that congenital decreases in serum IGF-1 levels (LID) associated with decreased lifespan in male mice and increased incidence of hepatic tumors, likely reflecting lifelong increases in GH secretion. In contrast to our results showing no change in female LID lifespan, Svensson *et al*. (2011) observed increased median lifespan of female LID mice with no significant difference in male LID lifespan compared with wild-type mice. In that study, LID mice also showed reduced body weight similar to what we observed. The reason(s) why our current results differ from Svensson *et al*. is unclear, but could be related to the different genetic backgrounds between the control and LID mice in our study. However, it is also worth noting that the control mice in our study had robust median lifespan of over 30 months, whereas the control mice in the Svensson *et al*. study had median lifespans of 22-23 months, which is shorter than expected for C57BL/6 strain. Thus, the LID female mice in the Svensson study may have been protected against some life-shortening stimulus. Likewise, in longevity studies (conducted at three different locations) with mice homozygous for an insertion in the *Igf1* gene, which leads to significant reductions in IGF-1 levels, it was found that the maximum lifespan significantly increased and age-specific mortality rates were reduced in the IGF-1-deficient mice (Lorenzini *et al*., 2013). However, mean lifespan was increased in female IGF-1-deficient animals in only one site and early life mortality was noted in one cohort of IGF-1-deficient mice, indicating that reductions in IGF-1 alone are insufficient to increase both mean and maximal lifespan in mice.

Using the iLID model, we induced IGF-1 deficiency after the critical phase of growth and development and followed the mice up to two years of age. We have found that in iLID mice reductions in serum IGF-1 and concomitant increases in GH levels during aging associated with increased liver weight, systemic and liver inflammation, increased oxidative stress, and increased incidence of hepatic tumors, although this did not reach significance. We postulate that this phenotype relates to the unopposed action of high GH levels on hepatocytes, as evidenced by the increased basal STAT5b phosphorylation in livers from iLID mice. Possible increase in hepatic GH action was also evident by increase in hepatocyte nuclear size in the iLID mice. These data are in accordance with previous observations using the GH transgenic mice (Miquet *et al*., 2013), which also manifested increased hepatocellular nuclear size. Using the iLID model, we show, for the first time, that elevations in GH late in life have detrimental effects on the liver.

Inducible liver IGF-1-deficient mice showed increased macrophage infiltration and other cellular infiltrate in the liver. GH has been implicated in recruitment of inflammatory cells (Franceschi *et al*., 2005), suggesting that the excess of GH action in the liver of the iLID mice triggered hepatic inflammation. In GH-deficient (GHD) patients, who exhibit major reductions in serum IGF-1, there is significantly impairment of natural killer (NK) cell activity, both unstimulated and stimulated by INFγ or IL-2. Similarly, NK cell concentration and the proportion of NK cells (CD16+) were reduced in GHD patients compared with controls (Sneppen *et al*., 2002). On the other hand, an increase in T-cell activity and a decrease in B-cell activity have been demonstrated in a very large cohort of patients with acromegaly, with high levels of serum GH and IGF-1 (Colao *et al*., 2002). However, despite this valuable information, these studies demonstrate conditions in which the changes in GH and IGF-1 paralleled, namely both decreased as seen in states of GHD, or increased as seen in acromegaly. Thus, the relative contribution of each hormone to the inflammatory phenotype is hard to be determined. In the iLID model, we show that in the face of low levels of serum IGF-1, high levels of GH in serum associate with increased inflammation. These results strongly suggest causality between GH elevations and hepatic inflammation.

The aforementioned increases in hepatic inflammation, oxidative stress, and GHR activation in livers of the iLID mice were associated with increased incidence of hepatic tumors, although this did not reach significance. In LID mice, however, where serum IGF-1 decreased throughout lifespan, we have shown a significant increase in liver tumors. We attribute the increase in hepatic tumor incidence to the high levels of GH in the LID and iLID mice. Our findings are in line with previous observations showing a link between increased GH signaling and hepatic tumors (Baik *et al*., 2011; Mueller *et al*., 2011; Miquet *et al*., 2013). Accordingly, Sonntag *et al*. (2005) has shown that reductions in both GH and IGF-1 during adulthood and aging, reduced neoplastic disease, nephropathy, and total disease burden. Unfortunately, we did not perform survival studies in the iLID cohort, and thus, we cannot conclude that the findings above contributed to the overall mortality of the iLID mice.

Aged iLID mice demonstrated increased oxidative stress in liver and muscle. This was evident by significant increases in tissue levels of oxidative proteins in both livers and muscles of iLID mice as compared to controls. Our findings are consistent with recent studies demonstrating increased cellular oxidative stress in aged animals with adult onset IGF-1 deficiency (Bailey-Downs *et al*., 2012). In that study, cultured aorta segments of IGF-1-deficient mice treated with a panel of oxidative stressors showed attenuation in Nrf2-driven genes, leading to endothelial dysfunction, increased oxidative stress, and apoptosis. In line with these data, it has been shown that IGF-1 can oppose oxidative stress-induced damage in the vasculature by up-regulating glutathione peroxidase (Higashi *et al*., 2013). Consistent with this evidence, we have found that fibroblasts isolated from aged iLID mice show increased sensitivity to peroxide, as reflected by increased levels of cleaved PARP. We postulate that the increased sensitivity to oxidative stress is due to increases in serum GH levels during aging. It has been previously shown that fibroblast cultures from aged GH-deficient Snell, Ames, and GHRKO mice were resistant to the cytotoxic effects of peroxide and paraquat (Murakami *et al*., 2003; Salmon *et al*., 2005). In Snell dwarfs, resistance to peroxide and paraquat was associated with increased catalase activity (Page *et al*., 2009).

Lastly, we studied bone loss, another physiological hallmark of aging. In humans, the physiological decline in GH and IGF-1 levels is linked to osteoporosis. Using the iLID model, we were able to delineate the role of the GH/IGF-1 axis in skeletal aging, without the confounding effects of this axis on the growing skeleton. In our previous studies characterizing the LID mice during development, we found that reduced serum levels of IGF-1 in LID mice were associated with the development of slender bones (Elis *et al*., 2010a). This slender phenotype developed at an early pubertal age and persisted to 52 weeks, affecting mostly radial bone growth and minimally affecting linear growth. In a subsequent study, we performed skeletal characterization of the iLID mice, where depletion of serum IGF-1 was induced at 4 (prepuberty) and at 8 (postpuberty) weeks of age (Courtland *et al*., 2011). We showed that depletion of serum IGF-1 during growth compromised peak bone mass. Here, we depleted serum IGF-1 during advanced adulthood (iLID), when bone loss becomes more prevalent, and we showed that reductions in serum IGF-1 during aging impaired bone integrity. Taken together, data from both studies show that depletion of serum IGF-1 during growth affects skeletal development and peak bone accrual, while during aging, serum IGF-1 appears to play protective roles on the skeleton and its reductions affect mainly the cortical compartment.

In conclusion, animal and human studies have revealed major roles of the GH/IGF-1 axis in the regulation of growth, development, and organ function. As such, decline in these two pleiotropic hormones during aging was shown to associate with organ dysfunction. On the other hand, high/normal levels of GH/IGF-1 during aging have been associated with the development of neoplastic disease in both human and animals, and complete lifelong suppression of GH signaling in rodents produces robust extension of longevity. Thus, the involvement of this axis in the aging phenotype remains controversial, and the relative contribution of each hormone (GH or IGF-1) to age-related pathology is still doubtful and may be tissue specific. We have shown here that low circulating levels of IGF-1 coupled with high levels of GH during aging, associate with hepatomegaly and increased hepatic pathology in both LID and iLID models and with reduced longevity of LID males. At the same time, we show that elevations in GH were insufficient to protect against bone loss during aging.

## Methods

### Animals

The iLID model (C57BL/6 background) has been described previously (Courtland *et al*., 2011). Male mice used in this study were given unrestricted access to water and food and housed to a maximum of 5 per cage under a 12 h light/dark cycle. Animal care and maintenance were provided through the NYU AAALAC Accredited Animal Facility. All procedures approved by the Institutional Animal Care and Use Committee of the NYU. See additional details in supplemental methods.

Lifespan studies of LID mice on a mixed genetic background (C57BL/6, FVB/N, and 129sv) were conducted in the Nathan Shock Center at Texas Health Center, San Antonio (UTHSCSA). Food intake was also recorded. Pathological lesions were identified and graded for severity as previously described (Ikeno *et al*., 2005). See additional details in Methods S1.

Animal care and maintenance were provided through the UTHSCSA AAALAC Accredited Animal Facility. All procedures approved by the Institutional Animal Care and Use Committee of the UTHSCSA.

### Hormone measurements

Mice were bled between 7 and 9 AM via the mandibular vein before sacrifice. Serum samples were collected in a fed state at 22-24 months of age. Serum IGF-1 levels were measured by RIA (American Laboratory Products Company, Inc., Salem, NH, USA). Serum GH levels were determined using commercial ELISA kit (Millipore, Temecula, CA, USA). Serum osteocalcin levels were measured by ELISA (Millipore Inc). Serum PTH levels were determined by mouse PTH ELISA (Immutopics Inc., San Clemente, CA, USA).

### Serum isoprostanes

8-iso PGF2a levels were measured by GC/MS as described by Roberts and Morrow (Roberts & Morrow, 2000). The 8-iso PGF2a concentrations are computed using either peak area or peak height, that is, area of sample/area of standard x ng standard added = ng 8-iso PGF2a in the sample. Data were normalized to plasma volume.

### Serum cytokine assay

Serum cytokine levels were assessed using mouse proinflammatory 7-plex ultra-sensitive kit (K15013C-1, Meso Scale Discovery) according to the manufacturer’s instructions. Plates were read on a SECTOR plate reader.

### Gene expression

Total RNA was extracted from tissues using TRIzol (Invitrogen, Carlsbad, CA). RNA samples (1 μg) were reverse-transcribed using oligo-(dT) primers (Invitrogen), and quantitative real-time PCR was performed following the manufacturer’s instructions using the QuantiTect™ SYBR® green PCR kit (Qiagen, Valencia, CA, USA) on an ABI PRISM 7900HT sequence detection system (Applied Biosystems, Foster City, CA, USA). Transcript levels were assayed 3 times in each sample, and the fold-change ratios between experimental and control samples were calculated. We used three housekeeping genes; β-actin, GAPDH, and 18S. As no differences were found when data corrected to each housekeeping gene, we present data obtained after correction to 18S. For primer sequences, refer to Table S6.

### Tissue oxidative stress levels

oxidative stress was measured using OxyBlot Protein Oxidation Detection Kit (S7150, Millipore). Proteins extracts obtained in RIPA buffer (25 mm Tris-HCl pH 7.6, 150 mm NaCl, 1% Triton X-100, 1% sodium deoxycholate, 0.1% SDS). 20 µg of protein extracts from liver and muscles of control and iLID mice were analyzed according to the manufacturer’s instructions.

### Primary fibroblast cultures

One centimeter of mouse tail was rinsed in 70% ethanol three times and consequently with PBS to remove excess alcohol. Tails were minced with a sterile scalpel (#22 Blade, Fisher, 08-918-5C; Scalpel Handle, 08-917-5) and digested overnight with collagenase (400 U mL^−1^, Gibco, 17101-015). Cells were then passed through a sterile nylon netting (40-μm nylon mesh, BD Falcon, 25-2340 with Falcon 50 mL culture tubes, 35-2098) to remove small pieces of skin and hair. Cells were counted and seeded at 0.25 × 10^6^ cells in 6 mL complete DMEM in a T-25 tissue culture flask. To evaluate oxidative stress resistance, cells were seeded in 6-well plates and treated with 400 μm peroxide for 8 or 24 h. At the end of treatment, cells were harvested in RIPA buffer (25 mm Tris-HCl pH 7.6, 150 mm NaCl, 1% Triton X-100, 1% sodium deoxycholate, 0.1% SDS). Twenty microgram of total protein extracts was run on 4-10% SDS-PAGE, and PARP (#9542, Cell Signaling, Danvers, MA, USA) cleavage was detected by immunoblot. The levels of cleaved PARP were corrected to both total PARP and β-actin (Cell Signaling, #4970), detected on the same blot.

### Tissue protein extraction and detection

Liver proteins were obtained from 50 mg liver tissue homogenized in RIPA buffer (25 mm Tris-HCl pH 7.6, 150 mm NaCl, 1% Triton X-100, 1% sodium deoxycholate, 0.1% SDS). Twenty microgram of total protein extracts of control and iLID mice were run on 4-20% SDS-PAGE. Membranes were blocked in 5% nonfat dry milk and subjected to immunoblot. Phospho-STAT5b (Cell Signaling, CAT # 9359) and total STAT5b (Cell Signaling, CAT# 9363) were diluted in TBS-T (1:1000) and detected according to manufacturer’s instructions. β-actin (Cell Signaling, CAT# 4970) was used as a loading control. Levels of phosphorylated STAT5b were corrected to total STAT5b, using a stripped membrane. Quantification was performed by FUJIFilm LAS3000, and 10 mice from each group were used for the quantification.

### Primary osteoblast-like cultures

Bone marrow cells were flushed out from femurs using a 26-gauge needle and collected in αMEM culture medium and drawn through 18-gauge needle to achieve single-cell suspension. Cells were then washed in αMEM culture medium and cultured in αMEM supplemented with 10% heat-inactivated FBS with β-glycerophosphate (10 mm) and ascorbic acid (50 mm). Cellular alkaline phosphatase activity was determined using a para-nitrophenol phosphate-based colorimetric assay. Cultures were carried out for different time points as indicated. Alkaline phosphate (ALP) staining was performed using an Alkaline Phosphatase kit according to the manufacturer’s instructions (Sigma-Aldrich, St. Louis, MO, USA).

### Histology

Tissues were fixed in 10% buffered formalin phosphate (Fisher Scientific) and embedded in paraffin. Immunohistochemistry with rabbit anti-mouse Iba1 (Wako Chemicals, VA) was performed in 5 um sections of paraffin-embedded livers. Three sections were analyzed per mouse, where the percentage of Iba-1-positive cells was calculated per each section. Nuclear size was evaluated in H&E-stained liver sections. We analyzed one section per mouse and 5 mice per group that were blinded to the observer. Fifty nuclei per section were measured and calibrated to an image scale bar using Image J software (National Institutes of Health, Bethesda, MD, USA).

### Micro-CT

Femoral bone morphology at the mid-diaphysis, and trabecular bone volume fraction and micro-architecture in the distal femoral metaphysis were assessed as previously described (Elis *et al*., 2010a,b) and according to JBMR guidelines (Bouxsein *et al*., 2010). See additional details in Methods S1.

### Micro-Positron Emission Tomography/Computed Tomography

Positron emission tomography scan was performed in a Micro-Positron Emission Tomography/Computed Tomography (μ-PET/CT) (Siemens, TN, USA) using 18F-fluorodeoxyglucose (18F-FDG). The animals were fasted for 8-10 h before undergoing PET. They were anesthetized using 1 L min^−1^ of 2% isoflurane in 100% oxygen and injected intravenously with 10 MBq 18F-FDG. After 1 h had been allowed for clearance of nonspecifically bound tracer, the animals received an intraperitoneal injection of 30 μL of a mixture of ketamine and xylazine (80 and 20 mg mL^−1^, respectively) and subsequently imaged. Static acquisition was performed for 30 min.

### Mechanical testing by 4-point bending assay

Mouse femora from 2-year-old control and iLID mice were tested to failure by 4-point bending using a servohydraulic materials testing system (Bose, Canton, MA, USA). This test measures whole-bone stiffness, maximum load, postyield deflection and work to failure. Femora were placed with the anterior surface down on two lower supports. The two lower and two upper supports were set apart by 6.35 and 2.2 mm, respectively. Loading was centered over the mid-shaft, at a displacement of 0.05 mm s^−1^ until failure.

### Inductively coupled plasma

Thermo Jarrell Ash Trace Scan Advantage was used to determine bone mineral compositions (Ca,P). 10 mg of bone mineral powder was dissolved in 17% HCl and made up to 100 mL in a volumetric flask with double distilled water. Appropriate standard solutions for Ca (0, 1 and 50 ppm) and P (0, 1 and 30 ppm) were prepared from 100 ppm standard solutions of the respective elements (Fisher Scientific). The specimen in solution and standard solutions was pumped through argon plasma excited by 2 kW 27.12 MHz radio frequency generator. The concentrations of each element were determined using their characteristic wavelengths (Ca: 317.9Å, P: 213.6 Å).

### Statistical analysis

For survival studies of the LID mice and end-of-life pathology, total frequency and grade of lesions were compared between genotypes using a chi-square test. When the expected frequencies were too small for the chi-square test, the data were analyzed using Fisher’s exact test. The Kaplan–Meier survival curves for fatal neoplastic lesions and adenocarcinoma were analyzed using a log-rank test (Lawless 1990). All differences in mean serum hormone levels, organ weights, and growth between controls and iLID mice were assessed by T-test. Values are presented as the mean ± SEM, and *P* < 0.05 was considered statistically significant. All micro-CT measurements, mechanical tests, and ICP data are presented as the mean ± SD.

## Supporting Information

**Methods S1** Reductions in serum IGF-1 during aging impair health span. **Table S1** Support information of survival studies with the congenital LID mouse cohort. **Table S2** Body and organ weights of a subcohort of control and LID mice dissected at 19-20 months of age. **Table S3** Body and organ weights of 2 year-old control and iLID mice. **Table S4** Cytokine levels in serum assessed by Mouse pro-inflammatory 7-plex ultra-sensitive kit. **Table S5** Tumor incidence in control and iLID mice. **Table S6** Primers used for gene expression studies using real-time PCR.

## References

[b1] Baik M, Yu JH, Hennighausen L (2011). Growth hormone-STAT5 regulation of growth, hepatocellular carcinoma, and liver metabolism. Ann. N. Y. Acad. Sci.

[b2] Bailey-Downs LC, Mitschelen M, Sosnowska D, Toth P, Pinto JT, Ballabh P, Valcarcel-Ares MN, Farley J, Koller A, Henthorn JC, Bass C, Sonntag WE, Ungvari Z, Csiszar A (2012). Liver-specific knockdown of IGF-1 decreases vascular oxidative stress resistance by impairing the Nrf2-dependent antioxidant response: a novel model of vascular aging. J. Gerontol. A Biol. Sci. Med. Sci.

[b3] Bartke A, Chandrashekar V, Bailey B, Zaczek D, Turyn D (2002). Consequences of growth hormone (GH) overexpression and GH resistance. Neuropeptides.

[b4] Bartke A, Sun LY, Longo V (2013). Somatotropic signaling: trade-offs between growth, reproductive development, and longevity. Physiol. Rev.

[b5] Barzilai N, Huffman DM, Muzumdar RH, Bartke A (2012). The critical role of metabolic pathways in aging. Diabetes.

[b6] Bokov AF, Garg N, Ikeno Y, Thakur S, Musi N, DeFronzo RA, Zhang N, Erickson RC, Gelfond J, Hubbard GB, Adamo ML, Richardson A (2011). Does reduced IGF-1R signaling in Igf1r+/- mice alter aging?. PLoS ONE.

[b7] Bouxsein ML, Boyd SK, Christiansen BA, Guldberg RE, Jepsen KJ, Muller R (2010). Guidelines for assessment of bone microstructure in rodents using micro-computed tomography. J. Bone Miner. Res.

[b8] Colao A, Ferone D, Marzullo P, Panza N, Pivonello R, Orio F, Grande G, Bevilacqua N, Lombardi G (2002). Lymphocyte subset pattern in acromegaly. J. Endocrinol. Invest.

[b9] Colman RJ, Anderson RM, Johnson SC, Kastman EK, Kosmatka KJ, Beasley TM, Allison DB, Cruzen C, Simmons HA, Kemnitz JW, Weindruch R (2009). Caloric restriction delays disease onset and mortality in rhesus monkeys. Science.

[b10] Courtland HW, Elis S, Wu Y, Sun H, Rosen CJ, Jepsen KJ, Yakar S (2011). Serum IGF-1 affects skeletal acquisition in a temporal and compartment-specific manner. PLoS ONE.

[b11] Elis S, Courtland HW, Wu Y, Rosen CJ, Sun H, Jepsen KJ, Majeska RJ, Yakar S (2010a). Elevated serum levels of IGF-1 are sufficient to establish normal body size and skeletal properties even in the absence of tissue IGF-1. J. Bone Miner. Res.

[b12] Elis S, Courtland HW, Wu Y, Fritton JC, Sun H, Rosen CJ, Yakar S (2010b). Elevated serum IGF-1 levels synergize PTH action on the skeleton only when the tissue IGF-1 axis is intact. J. Bone Miner. Res.

[b13] Franceschi C, Olivieri F, Marchegiani F, Cardelli M, Cavallone L, Capri M, Salvioli S, Valensin S, De Benedictis G, Di Iorio A, Caruso C, Paolisso G, Monti D (2005). Genes involved in immune response/inflammation, IGF1/insulin pathway and response to oxidative stress play a major role in the genetics of human longevity: the lesson of centenarians. Mech. Ageing Dev.

[b14] Guevara-Aguirre J, Balasubramanian P, Guevara-Aguirre M, Wei M, Madia F, Cheng CW, Hwang D, Martin-Montalvo A, Saavedra J, Ingles S, de CaboR, Cohen P, Longo VD (2011). Growth hormone receptor deficiency is associated with a major reduction in pro-aging signaling, cancer, and diabetes in humans. Sci. Transl. Med.

[b15] Hauck SJ, Bartke A (2000). Effects of growth hormone on hypothalamic catalase and Cu/Zn superoxide dismutase. Free Radic. Biol. Med.

[b16] Higashi Y, Pandey A, Goodwin B, Delafontaine P (2013). Insulin-like growth factor-1 regulates glutathione peroxidase expression and activity in vascular endothelial cells: Implications for atheroprotective actions of insulin-like growth factor-1. Biochim. Biophys. Acta.

[b17] Holzenberger M, Dupont J, Ducos B, Leneuve P, Geloen A, Even PC, Cervera P, Le Bouc Y (2003). IGF-1 receptor regulates lifespan and resistance to oxidative stress in mice. Nature.

[b18] Ikeno Y, Hubbard GB, Lee S, Richardson A, Strong R, Diaz V, Nelson JF (2005). Housing density does not influence the longevity effect of calorie restriction. J. Gerontol. A Biol. Sci. Med. Sci.

[b20] Kappeler L, De Magalhaes Filho C, Dupont J, Leneuve P, Cervera P, Perin L, Loudes C, Blaise A, Klein R, Epelbaum J, Le Bouc Y, Holzenberger M (2008). Brain IGF-1 receptors control mammalian growth and lifespan through a neuroendocrine mechanism. PLoS Biol.

[b21] Laron Z (2005). Do deficiencies in growth hormone and insulin-like growth factor-1 (IGF-1) shorten or prolong longevity?. Mech. Ageing Dev.

[b201] Lawless JF, Jerald F Lawless (1990). Basic Concepts and Models. Statistical models and methods for lifetime data.

[b22] Lombardi G, Tauchmanova L, Di Somma C, Musella T, Rota F, Savanelli MC, Colao A (2005). Somatopause: dismetabolic and bone effects. J. Endocrinol. Invest.

[b23] Lorenzini A, Salmon AB, Lerner C, Torres C, Ikeno Y, Motch S, McCarter R, Sell C (2013). Mice Producing Reduced Levels of Insulin-Like Growth Factor Type 1 Display an Increase in Maximum, but not Mean, Life Span. J Gerontol A Biol Sci Med Sci.

[b24] Mattison JA, Roth GS, Beasley TM, Tilmont EM, Handy AM, Herbert RL, Longo DL, Allison DB, Young JE, Bryant M, Barnard D, Ward WF, Qi W, Ingram DK, de Cabo R (2012). Impact of caloric restriction on health and survival in rhesus monkeys from the NIA study. Nature.

[b25] Miquet JG, Freund T, Martinez CS, Gonzalez L, Diaz ME, Micucci GP, Zotta E, Boparai RK, Bartke A, Turyn D, Sotelo AI (2013). Hepatocellular alterations and dysregulation of oncogenic pathways in the liver of transgenic mice overexpressing growth hormone. Cell Cycle.

[b26] Mueller KM, Kornfeld JW, Friedbichler K, Blaas L, Egger G, Esterbauer H, Hasselblatt P, Schlederer M, Haindl S, Wagner KU, Engblom D, Haemmerle G, Kratky D, Sexl V, Kenner L, Kozlov AV, Terracciano L, Zechner R, Schuetz G, Casanova E, Pospisilik JA, Heim MH, Moriggl R (2011). Impairment of hepatic growth hormone and glucocorticoid receptor signaling causes steatosis and hepatocellular carcinoma in mice. Hepatology.

[b27] Murakami S, Salmon A, Miller RA, Faseb J (2003). Multiplex stress resistance in cells from long-lived dwarf mice. Faseb J.

[b28] O’Neill C, Kiely AP, Coakley MF, Manning S, Long-Smith CM (2012). Insulin and IGF-1 signalling: longevity, protein homoeostasis and Alzheimer’s disease. Biochem. Soc. Trans.

[b29] Page MM, Salmon AB, Leiser SF, Robb EL, Brown MF, Miller RA, Stuart JA (2009). Mechanisms of stress resistance in Snell dwarf mouse fibroblasts: enhanced antioxidant and DNA base excision repair capacity, but no differences in mitochondrial metabolism. Free Radic. Biol. Med.

[b30] Roberts LJ, Morrow JD (2000). Measurement of F(2)-isoprostanes as an index of oxidative stress in vivo. Free Radic. Biol. Med.

[b31] Salmon AB, Murakami S, Bartke A, Kopchick J, Yasumura K, Miller RA (2005). Fibroblast cell lines from young adult mice of long-lived mutant strains are resistant to multiple forms of stress. Am. J. Physiol. Endocrinol. Metab.

[b32] Selman C, Lingard S, Choudhury AI, Batterham RL, Claret M, Clements M, Ramadani F, Okkenhaug K, Schuster E, Blanc E, Piper MD, Al-Qassab H, Speakman JR, Carmignac D, Robinson IC, Thornton JM, Gems D, Partridge L, Withers DJ (2008). Evidence for lifespan extension and delayed age-related biomarkers in insulin receptor substrate 1 null mice. Faseb J.

[b33] Sneppen SB, Mersebach H, Ullum H, Feldt-Rasmussen U (2002). Immune function during GH treatment in GH-deficient adults: an 18-month randomized, placebo-controlled, double-blinded trial. Clin. Endocrinol (Oxf).

[b34] Sonntag WE, Carter CS, Ikeno Y, Ekenstedt K, Carlson CS, Loeser RF, Chakrabarty S, Lee S, Bennett C, Ingram R, Moore T, Ramsey M (2005). Adult-onset growth hormone and insulin-like growth factor I deficiency reduces neoplastic disease, modifies age-related pathology, and increases life span. Endocrinology.

[b35] Sonntag WE, Csiszar A, deCabo R, Ferrucci L, Ungvari Z (2012). Diverse roles of growth hormone and insulin-like growth factor-1 in mammalian aging: progress and controversies. J. Gerontol. A Biol. Sci. Med. Sci.

[b36] Stein PK, Soare A, Meyer TE, Cangemi R, Holloszy JO, Fontana L (2012). Caloric restriction may reverse age-related autonomic decline in humans. Aging Cell.

[b37] Svensson J, Sjogren K, Faldt J, Andersson N, Isaksson O, Jansson JO, Ohlsson C (2011). Liver-derived IGF-I regulates mean life span in mice. PLoS ONE.

[b38] Taguchi A, Wartschow LM, White MF (2007). Brain IRS2 signaling coordinates life span and nutrient homeostasis. Science.

[b39] Ward WF, Qi W, Van Remmen H, Zackert WE, Roberts LJ, Richardson A (2005). Effects of age and caloric restriction on lipid peroxidation: measurement of oxidative stress by F2-isoprostane levels. J. Gerontol. A Biol. Sci. Med. Sci.

[b40] Westbrook R, Bonkowski MS, Strader AD, Bartke A (2009). Alterations in oxygen consumption, respiratory quotient, and heat production in long-lived GHRKO and Ames dwarf mice, and short-lived bGH transgenic mice. J. Gerontol. A Biol. Sci. Med. Sci.

[b41] Wolf NS, Li Y, Pendergrass W, Schmeider C, Turturro A (2000). Normal mouse and rat strains as models for age-related cataract and the effect of caloric restriction on its development. Exp. Eye Res.

[b42] Wu Y, Brodt P, Sun H, Mejia W, Novosyadlyy R, Nunez N, Chen X, Mendoza A, Hong SH, Khanna C, Yakar S (2010). Insulin-like growth factor-I regulates the liver microenvironment in obese mice and promotes liver metastasis. Cancer Res.

[b43] Yakar S, Liu JL, Stannard B, Butler A, Accili D, Sauer B, LeRoith D (1999). Normal growth and development in the absence of hepatic insulin-like growth factor I. Proc. Natl. Acad. Sci. U.S.A.

