# Supplementary material for: Reductions in serum IGF-1 during aging impair health span
Source: Aging Cell. 2013 Dec 30;13(3):408–18. doi: 10.1111/acel.12188 (PMC4326899; doi:10.1111/acel.12188)
Supplement: Supplementary file 1 — Methods S1 Reductions in serum IGF-1 during aging impair health span. Table S1 Support information of survival studies with the congenital LID mouse cohort. Table S2 Body and organ weights of a subcohort of control and LID mice dissected at 19-20 months of age. Table S3 Body and organ weights of 2 year-old control and iLID mice. Table S4 Cytokine levels in serum assessed by Mouse pro-inflammatory 7-plex ultra-sensitive kit. Table S5 Tumor incidence in control and iLID mice. Table S6 Primers used for gene expression studies using real-time PCR. [file acel0013-0408-sd1.docx]

**Reductions in serum IGF-1 during aging impair health span.**

**Supplemental methods:**

**Animals**: The iLID model (C57BL/6 background) has been described previously ([21](#_ENREF_21)). Briefly, iLID was created using the Cre-loxP system and crossing mice with floxed exon 4 of the IGF-1 gene with transgenic mice expressing tamoxifen-inducible Cre-recombinase under the trypsin-1α promoter. In this model, iLID mice (which are homozygous for the floxed IGF-1 and carry the Cre-recombinase transgene) exhibit normal levels of IGF-1 in serum. Upon tamoxifen injection, an igf-1 gene recombination occurs specifically in hepatocytes, leading to ~65% reductions in serum IGF-1 levels. Control mice are homozygous for the floxed IGF-1 allele but do not carry the Cre-recombinase and therefore do not recombine the igf-1 gene in response to tamoxifen injection. Male mice used in this study were given unrestricted access to water and food, and housed to a maximum of 5 per cage under a 12 hours light:dark cycle. Animal care and maintenance were provided through the NYU AAALAC Accredited Animal Facility. All procedures approved by the Institutional Animal Care and Use Committee of the NYU.

Lifespan studies of LID mice on a mixed genetic background (C57BL/6, FVB/N and 129sv) were conducted in the Nathan Shock Center at Texas Health Center, San Antonio (UTHSCSA). Control mice had floxed but intact Igf1 alleles and were on the C57BL/6 and 129 background. Male and female mice were assigned to survival cohorts and followed until each mouse died. Mice were weighed at 2-week intervals. Food intake was also recorded. Each mouse in the survival cohort received an identifying microchip, which permitted accurate recording of food consumption and body weight. Full gross and histopathology was performed. Pathological lesions were identified and graded for severity as previously described ([39](#_ENREF_39)). Two pathologists separately examined all of the samples without knowledge of their genotype or age. The probable cause of death was determined independently by the two pathologists based on the severity of the pathology found at necropsy. In more than 90% of the cases, there was agreement by the two pathologists. In cases where the two pathologists did not agree or where disease did not appear severe enough, the cause of death was categorized as unknown.

Animal care and maintenance were provided through the UTHSCSA AAALAC Accredited Animal Facility. All procedures approved by the Institutional Animal Care and Use Committee of the UTHSCSA.

**Micro-CT**: Femoral bone morphology at the mid-diaphysis, and trabecular bone volume fraction and microarchitecture in the distal femoral metaphysis were assessed as previously described ([38](#_ENREF_38), [41](#_ENREF_41)) and according to JBMR guidelines ([42](#_ENREF_42)). Femora were reconstructed at 6.7micron voxel resolution. For trabecular bone regions, we assessed the bone volume fraction (BV/TV, %), trabecular thickness (Tb.Th, mm), trabecular number (Tb.N), and trabecular spacing (Tb.Sp, mm). Bone mineral density (BMD) was defined as the average mineral value of the bone voxels and expressed in hydroxyapatite density equivalents (HA g/cc). For cortical bone at the femoral mid-shaft, we measured the average total cross-sectional area inside the periosteal envelope (Tt.Ar, mm^2^), the cortical bone and medullary area within this same envelope (Ct.Ar, mm^2^ and Ma.Ar mm^2^, respectively), the relative cortical area (RCA; Ct.Ar/Tt.Ar), the average cortical thickness (Ct.Th, mm) and the polar moment of inertia (J_o_ mm^4^). Tissue mineral density (TMD) was defined as the average mineral value of the bone voxels *within* the cortical bone and expressed in hydroxyapatite density equivalents (HA g/cc). All regions of analysis were standardized according to anatomical landmarks.

**Supplemental Table 1:** Support information of survival studies with the congenital LID mouse cohort.

| Cage number | Mouse number | Genotype | Sex | DOB | DOD | Age, days | Average lifespan (Mean±SEM) |
| --- | --- | --- | --- | --- | --- | --- | --- |
| 1 | 1 | Control | M | 05/29/02 | 12/10/04 | 926 | 1014±55 days  34.7±1.3 months |
|  | 2 |  |  |  | 04/28/05 | 1065 |  |
|  | 3 |  |  |  | 04/20/05 | 1057 |  |
|  | 4 |  |  |  | 06/01/05 | 1099 |  |
| 2 | 1 | Control | M | 04/27/02 | 10/11/04 | 898 |  |
|  | 2 |  |  |  | 01/19/06 | 1363 |  |
|  | 3 |  |  |  | 10/13/04 | 900 |  |
|  | 4 |  |  |  | 04/30/04 | 734 |  |
| 3 | 1 | Control | M | 05/24/02 | 03/04/05 | 1015 |  |
|  | 2 |  |  |  | 05/19/05 | 1091 |  |
|  | 3 |  |  |  | 02/06/04 | 623 |  |
| 4 | 1 | Control | M | 04/04/02 | 10/11/04 | 921 |  |
|  | 2 |  |  |  | 09/08/05 | 1253 |  |
|  | 3 |  |  |  | 06/01/05 | 1154 |  |
| 5 | 1 | Control | M | 05/29/02 | 02/08/05 | 986 |  |
|  | 2 |  |  |  | 07/04/05 | 1132 |  |
| 6 | 1 | LID | M | 04/27/02 | 03/03/05 | 1041 | 836±50 days  27.9±1.7 months |
|  | 2 |  |  |  | 08/16/04 | 842 |  |
|  | 3 |  |  |  | 10/16/04 | 903 |  |
| 7 | 1 | LID | M | 04/27/02 | 01/24/04 | 637 |  |
|  | 2 |  |  |  | 02/19/04 | 663 |  |
| 8 | 1 | LID | M | 03/26/02 | 01/26/05 | 1037 |  |
|  | 2 |  |  |  | 03/01/04 | 706 |  |
|  | 3 |  |  |  | 11/23/04 | 973 |  |
| 9 | 1 | LID | M | 04/27/02 | 03/27/05 | 1065 |  |
|  | 2 |  |  |  | 11/15/04 | 933 |  |
|  | 3 |  |  |  | 05/10/04 | 744 |  |
| 10 | 1 | LID | M | 04/27/02 | 05/04/01 | 738 |  |
|  | 2 |  |  |  | 09/19/03 | 510 |  |
| 11 | 1 | LID | M | 04/27/02 | 12/18/03 | 600 |  |
| 12 | 1 | LID |  | 05/10/02 | 04/07/04 | 698 |  |
| 13 | 1 | LID |  | 04/27/02 | 10/27/05 | 1279 |  |
| 14 | 1 | Control | F | 12/03/01 | 01/10/05 | 1134 | 1043±30 days  34.8±1.0 months |
|  | 2 |  |  |  | 09/21/04 | 1023 |  |
|  | 3 |  |  |  | 12/26/04 | 1119 |  |
| 15 | 1 | Control | F | 12/03/01 | 09/19/04 | 1021 |  |
| 16 | 1 | Control | F | 12/11/01 | 08/25/04 | 988 |  |
|  | 2 |  |  |  | 09/28/04 | 1022 |  |
|  | 3 |  |  |  | 06/29/04 | 931 |  |
| 17 | 1 | Control | F | 12/11/01 | 07/15/04 | 947 |  |
|  | 2 |  |  |  | 08/03/04 | 966 |  |
| 18 | 1 | Control | F | 03/17/02 | 04/25/04 | 770 |  |
|  | 2 |  |  |  | 09/06/05 | 1269 |  |
|  | 3 |  |  |  | 04/19/04 | 764 |  |
| 19 | 1 | Control | F | 03/18/02 | 06/18/05 | 1188 |  |
|  | 2 |  |  |  | 07/07/04 | 842 |  |
|  | 3 |  |  |  | 11/20/05 | 1343 |  |
|  | 4 |  |  |  | 05/25/05 | 1164 |  |
| 20 | 1 | Control | F | 03/18/02 | 12/08/05 | 1361 |  |
|  | 2 |  |  |  | 01/31/05 | 1050 |  |
| 21 | 1 | Control | F | 03/18/02 | 10/29/04 | 956 |  |
|  | 2 |  |  |  | 10/26/04 | 953 |  |
| 22 | 1 | Control | F | 03/21/02 | 11/29/04 | 984 |  |
|  | 2 |  |  |  | 12/15/04 | 1000 |  |
|  | 3 |  |  |  | 02/21/05 | 1068 |  |
| 23 | 1 | Control | F | 03/21/02 | 03/09/05 | 1084 |  |
|  | 2 |  |  |  | 04/16/05 | 1122 |  |
| 24 | 1 | LID | F | 11/11/01 | 10/28/04 | 1082 | 1087±38 days  36.2±1.2 months |
|  | 2 |  |  |  | 07/07/04 | 969 |  |
|  | 3 |  |  |  | 12/06/04 | 1121 |  |
| 25 | 1 | LID | F | 11/11/01 | 05/15/05 | 1281 |  |
|  | 2 |  |  |  | 01/05/05 | 1151 |  |
|  | 3 |  |  |  | 06/30/05 | 1327 |  |
| 26 | 1 | LID | F | 11/11/01 | 03/12/05 | 1217 |  |
|  | 2 |  |  |  | 04/16/05 | 1252 |  |
|  | 3 |  |  |  | 04/19/04 | 890 |  |
| 27 | 1 | LID | F | 03/18/02 | 11/22/04 | 980 |  |
|  | 2 |  |  |  | 11/17/04 | 975 |  |
| 28 | 1 | LID | F | 03/19/02 | 01/24/05 | 1042 |  |
|  | 2 |  |  |  | 11/01/04 | 958 |  |
|  | 3 |  |  |  | 05/10/04 | 783 |  |
|  | 4 |  |  |  | 12/31/04 | 1018 |  |
| 29 | 1 | LID | F | 03/26/02 | 09/03/05 | 1257 |  |
|  | 2 |  |  |  | 06/10/05 | 1172 |  |

**Supplemental Table 2:** Body and organ weights of a sub-cohort of control and LID mice dissected at 19-20 months of age. FHL- flexor hallucis longus, EDL-extensor digitorum longus.

|  | Control (n=7) | LID (n=9) | P |
| --- | --- | --- | --- |
| Body weight at 19-20 months, g | 29.61±3.38 | 28.54±3.15 | 0.52 |
| Liver, g  (%of body weight) | 1.25±0.19  (4.27±0.79) | 1.85±0.52  (6.45±1.70) | 0.01  0.00 |
| Kidney, g  (%of body weight) | 0.35±0.04  (1.20±0.19) | 0.27±0.04  (0.98±0.12) | 0.00  0.01 |
| Spleen, g  (%of body weight) | 0.06±0.01  (0.20±0.03) | 0.06±0.02  (0.22±0.09) | 0.84  0.68 |
| Heart, g  (%of body weight) | 0.13±0.00  (0.46±0.06) | 0.14±0.02  (0.51±0.06) | 0.21  0.11 |
| Lung, g  (%of body weight) | 0.16±0.05  (0.54±0.18) | 0.16±0.01  (0.58±0.09) | 0.82  0.58 |
| Muscle, mg:  Gastrocneius (%of body weight)  Plantaris (%of body weight)  FHL (%of body weight)  Soleus (%of body weight)  Quadriceps (%of body weight)  EDL+Tibialis (%of body weight) | 94.07±10.11 (0.32±0.05)  13.16±1.22 (0.04±0.00)  28.39±1.99 (0.09±0.00)  5.52±0.90 (0.02±0.00)  128.66±13.14 (0.43±0.04)  68.16±4.09 (0.23±0.02) | 90.22±10.40 (0.31±0.03)  10.89±1.36 (0.04±0.00)  25.88±3.28 (0.09±0.01)  5.17±0.79 (0.02±0.00)  113.99±14.38 (0.40±0.04)  61.67±8.43 (0.21±0.03) | 0.30 (0.77)  0.00 (0.00)  0.01 (0.16)  0.25 (0.65)  0.00 (0.03)  0.03 (0.10) |

**Supplemental Table 3:** Body and organ weights of 2 year-old control and iLID mice.

|  | Control (n=27) | iLID (n=36) | P |
| --- | --- | --- | --- |
| Body weight at 2 years old, g | 32.59±5.67 | 28.89±4.35* | 0.01 |
| Body length at 2 years old, cm | 10.12±0.59 | 9.81±0.48 | 0.10 |
| Liver, g  (%of body weight) | 1.78±0.63 (5.39±1.86) | 2.12±1.06 (7.44±3.69)* | 0.18  0.02 |
| Muscle, g  (%of body weight) | 0.24±0.03 (0.74±0.14) | 0.22±0.04 (0.77±0.12) | 0.13  0.36 |
| Gonadal fat, g  (%of body weight) | 1.26±0.84 (3.59±2.11) | 0.83±0.55* (2.66±1.61) | 0.03  0.06 |
| S.C fat, g  (%of body weight) | 0.52±0.32 (1.52±0.80) | 0.43±0.40 (1.35±1.05) | 0.34  0.50 |
| Kidney, g  (%of body weight) | 0.49±0.08 (1.52±0.24) | 0.39±0.06* (1.37±0.17)* | 0.00  0.01 |
| Spleen, g  (%of body weight) | 0.11±0.05 (0.34±0.17) | 0.09±0.05 (0.33±0.16) | 0.21  0.69 |
| Heart, g  (%of body weight) | 0.17±0.04 (0.52±0.09) | 0.17±0.04 (0.58±0.11)* | 0.84  0.05 |
| Lung, g  (%of body weight) | 0.27±0.23 (0.68±0.07) | 0.21±0.05 (0.71±0.14) | 0.18  0.32 |

**Supplemental Table 4:** Cytokine levels in serum assessed by Mouse pro-inflammatory 7-plex ultra-sensitive kit.

| Mice with microscopic tumors or undetectable tumors | | | |
| --- | --- | --- | --- |
| Cytokine, pg/ml | Control (n=11) | iLID (n=15) | *P* |
| INFγ | 2.86±0.99 | 2.84±0.50 | 0.98 |
| IL10 | 26.05±4.55 | 34.19±5.11 | 0.29 |
| IL12 | 41.99±11.47 | 59.13±22.14 | 0.61 |
| IL18 | 3.73±0.62 | 7.23±1.91 | 0.18 |
| IL6 | 56.03±15.73 | 67.60±9.82 | 0.60 |
| KC/GRO | 121.97±11.33 | 125.05±15.20 | 0.89 |
| TNFα | 1.37±0.35 | 2.30±0.67 | 0.28 |
| Mice with macroscopic tumors | | | |
|  | N=6 | N=9 |  |
| INFγ | 2.89±0.76 | 2.88±0.76 | 0.99 |
| IL10 | 51.68±3.82 | 62.32±22.04 | 0.70 |
| IL12 | 40.27±22.52 | 66.55±14.68 | 0.32 |
| IL18 | 5.30±0.95 | 5.32±1.13 | 0.99 |
| IL6 | 62.60±19.00 | 205.74±144.36 | 0.44 |
| KC/GRO | 125.63±11.73 | 181.11±51.77 | 0.40 |
| TNFα | 2.10±0.87 | 2.18±0.49 | 0.93 |
| Inclusion of all mice of the same genotype | | | |
|  | N=17 | N=24 |  |
| INFγ | 2.73±0.58 | 2.86±0.41 | 0.98 |
| IL10 | 45.05±7.73 | 44.74±9.02 | 0.48 |
| IL12 | 42.75±9.68 | 62.31±13.98 | 0.32 |
| IL18 | 4.28±0.54 | 6.52±1.26 | 0.20 |
| IL6 | 79.79±20.30 | 119.40±54.30 | 0.39 |
| KC/GRO | 134.53±12.59 | 146.07±21.66 | 0.43 |
| TNFα | 1.66±0.33 | 2.25±0.43 | 0.32 |

(INFγ, Interferon γ; IL-, interleukin; KC/GRO-, keratinocyte chemoattractant, keratinocyte-derived chemokine/growth related oncogene; TNFα−,tumor necrosis-factor α). Data presented as Mean±SEM.

**Supplemental Table 5:** Tumor incidence in control and iLID mice.

|  | Control (n=27) | iLID (n=36) |
| --- | --- | --- |
| Number of mice with tumors (%) | 10/27 (37.04%) | 16/36 (44.44%) |
| Number of mice with hepatic tumors (%) | 7/27 (25.93%) | 15/36 (41.67%) |
| Number of mice that died prior to 2 years old (%) | 2/27 (7.40%) | 1/36 (2.77%) |

**Supplemental Table 6:** Primers used for gene expression studies using real-time PCR.

| Gene | Sequence-Forward | Sequence-Reverse |
| --- | --- | --- |
| Igf-1 | 5`-GGACCAGAGACCCTTTGCGGGG | 5`-GGCTGCTTTTGTAGGCTTCAGGTGG |
| IL-6 | 5`-TCTGCAAGAGACTTCCATCC | 5`-TTAGCCACTCCTTCTGTGAC |
| IL-1β | 5`-AAATACCTGTGGCCTTGGGC | 5`-CTTGGGATCCACACTCTCCAG |
| TNFα | 5`-GAACTGGCAGAAGAGGCACT | 5`-GGTCTGGGCCATAGAACTGA |
| F4/80 | 5`-AAGCATCCGAGACACACACAGTCT | 5`-TGACTGTACCCACATGGCTGATGA |
| Nrf2 (set 1) | 5`-CTCAGCATGATGGACTTGGAG | 5`-CACTTCTCGACTTACTCCAAGAT |
| Nrf2 (set 2) | 5`-AGCTACTCCCAGGTTGCCCAC | 5`-CAGGGCAAGCGACTCATGGTC |
| Gpx 1 | 5`-GTGGTGCTCGGTTTCCCGTGC | 5`-CCCGCCACCAGGTCGGACGTA |
| Catalaze 2 | 5`-GCTCGAGTGGCCAACTACCAG | 5`-TGAGCGCTGCTGCTCTGGTGC |
| MnSOD | 5`-CTGGCCAAGGGAGATGTTACA | 5`-GTCACGCTTGATAGCCTCCAG |
| β-actin | 5`-TGAACCCTAAGGCCAACCGTG | 5`-ATACAGGGACAGCACAGCCTG |
| GAPDH | 5`-TTGTGCAGTGCCAGCCTCGTC | 5`-GCGCCCAATACGGCCAAATCC |
| 18S | 5`-TCTTCTGCAACCTCCAGCATC | 5`-GACATGGCAGCCATTGTTCC |
